# Developing and testing accelerated partner therapy for partner notification for people with genital *Chlamydia trachomatis* diagnosed in primary care: a pilot randomised controlled trial

**DOI:** 10.1136/sextrans-2014-051994

**Published:** 2015-05-27

**Authors:** Claudia S Estcourt, Lorna J Sutcliffe, Andrew Copas, Catherine H Mercer, Tracy E Roberts, Louise J Jackson, Merle Symonds, Laura Tickle, Pamela Muniina, Greta Rait, Anne M Johnson, Kazeem Aderogba, Sarah Creighton, Jackie A Cassell

**Affiliations:** 1 Blizard Institute, Barts and the London School of Medicine & Dentistry, Queen Mary University of London, London, UK; 2 Research Department of Infection & Population Health, University College London, London, UK; 3 Health Economics Unit, School of Population and Health Sciences, College of Medical and Dental Sciences, University of Birmingham, Birmingham, UK; 4 Barts Sexual Health Centre, St Bartholomew's Hospital, Barts Health NHS Trust, London, UK; 5 Department of Sexual Health, Eastbourne District General Hospital, East Sussex Healthcare NHS Trust, Eastbourne, UK; 6 Homerton Sexual Health Services, Homerton Hospital, London, UK; 7 Division of Primary Care & Public Health, Brighton & Sussex Medical School, University of Brighton, Brighton, UK

**Keywords:** CHLAMYDIA TRACHOMATIS, PRIMARY CARE, PARTNER NOTIFICATION, CLINICAL STI CARE, COMPEX INTERVENTIONS

## Abstract

**Background:**

Accelerated partner therapy (APT) is a promising partner notification (PN) intervention in specialist sexual health clinic attenders. To address its applicability in primary care, we undertook a pilot randomised controlled trial (RCT) of two APT models in community settings.

**Methods:**

Three-arm pilot RCT of two adjunct APT interventions: APTHotline (telephone assessment of partner(s) plus standard PN) and APTPharmacy (community pharmacist assessment of partner(s) plus routine PN), versus standard PN alone (patient referral). Index patients were women diagnosed with genital chlamydia in 12 general practices and three community contraception and sexual health (CASH) services in London and south coast of England, randomised between 1 September 2011 and 31 July 2013.

**Results:**

199 women described 339 male partners, of whom 313 were reported by the index as contactable. The proportions of contactable partners considered treated within 6 weeks of index diagnosis were APTHotline 39/111 (35%), APTPharmacy 46/100 (46%), standard patient referral 46/102 (45%). Among treated partners, 8/39 (21%) in APTHotline arm were treated via hotline and 14/46 (30%) in APTPharmacy arm were treated via pharmacy.

**Conclusions:**

The two novel primary care APT models were acceptable, feasible, compliant with regulations and capable of achieving acceptable outcomes within a pilot RCT but intervention uptake was low. Although addition of these interventions to standard PN did not result in a difference between arms, overall PN uptake was higher than previously reported in similar settings, probably as a result of introducing a formal evaluation. Recruitment to an individually randomised trial proved challenging and full evaluation will likely require service-level randomisation.

**Trial registration number:**

Registered UK Clinical Research Network Study Portfolio id number 10123.

## Introduction

Across England, reported diagnoses of genital *Chlamydia trachomatis* continue to rise^1^ with almost 60% of all chlamydia in people under 25 years diagnosed in community settings^1^ where partner notification (PN) services may not be routinely available. Improving the effectiveness of PN, the process of identifying exposed sex partners and offering them testing and treatment^2^ ^3^ may have a greater impact on control of chlamydia than widening access to testing.^4^ ^5^ However, the optimal PN strategy for people with chlamydial infection is unknown. A recent systematic review^3^ suggests that expedited partner therapy (EPT),^6^ in which the doctor provides the index patient antibiotics or a prescription to give to their sex partner is more effective than simple patient referral (verbal advice that the partner(s) should attend) in preventing index reinfection, and achieves treatment of a higher proportion of sex partners.^7^ However, although EPT did not appear to be more effective than enhanced patient referral (simple patient referral supplemented by written advice, longer verbal explanation and or internet resources) in preventing index reinfection, EPT did achieve a higher proportion of sex partners reported treated, although evidence is limited.^5^

UK prescribing guidance does not support EPT in this form as it does not include clinical assessment of the sex partner.^8^ Accelerated partner therapy (APT), an adaptation of EPT using health adviser telephone-led and pharmacist-led PN interventions, includes a clinical assessment of the sex partner, does meet UK prescribing guidance and has shown promise in specialist settings.^8–10^ Modelling studies suggest that APT could play an important role in reducing index reinfection and prevention of reproductive health consequences especially in women, by reducing the time to successful partner treatment.^5^ ^7^

PN is known to be problematic in primary care^11–14^ but achievable within a chlamydia screening trial.^15^ General practitioners (GPs) report barriers to effective PN including lack of knowledge, skills, time and organisational issues.^13^ ^16–18^ Although GPs appear to be broadly supportive of APT,^16^ it may not directly translate to primary care settings in which staff are less skilled in sexual health, and service users may be different from those attending specialist services.

We aimed at assessing the feasibility, acceptability and preliminary evidence of effectiveness of APT for women diagnosed with chlamydia in non-specialist settings, through a pilot randomised controlled trial (RCT) of APT in contrasting primary care services in England. An economic evaluation is reported in detail elsewhere.^19^

## Methods

We carried out a three-arm pilot individually-randomised controlled trial of two APT interventions: APTHotline (telephone assessment of partner(s) by a health advisor plus standard PN) and APTPharmacy (community pharmacist assessment of partner plus standard PN), versus standard PN alone (patient referral) with one-to-one allocation. We defined standard care as the method of PN in routine use within each service. In practice, this varied between simple and enhanced patient referral, depending on the service and healthcare professional involved.

Participants were women aged at least 16 years, diagnosed in primary care with genital *C. trachomatis* infection (index patients) and at least one untreated contactable male sex partner in the last 6 months. Exclusion criteria for index patients were known HIV positive status, co-infection with other sexually transmitted infections (STIs) and an inability to understand English. Exclusion criteria for male sex partners were symptoms of complicated infection, allergy or contraindications to azithromycin and an inability to understand English as this precludes a safe telephone clinical assessment.

The trial took place between 1 September 2011 and 31 July 2013 in inner East London (deprived, young, ethnically mixed population: six general practices, two community (non-specialist) contraception and sexual health (CASH) services, five pharmacies) and the south coast of England (non-metropolitan population: six general practices, one CASH service, nine pharmacies).

We chose general practices and CASH services with the highest chlamydia screening and positivity rates and selected pharmacies based on their proximity to good transport links, ensuring broad geographical coverage around each recruitment site. If services devolved management of positive chlamydia cases to their local National Chlamydia Screening Programme, we approached the relevant office.

Index patients were recruited by healthcare professionals (practice nurses, GPs and CASH service health advisers) trained in the study procedures. They identified patients at the time of treatment and sought consent to participate. Male sexual partners were recruited by research health advisers on the APTHotline, or community pharmacists (depending on randomisation) who explained the study and sought informed consent. People who did not accept the offer of the trial were managed according to the service's routine practice.

### Interventions

**Figure 1:**
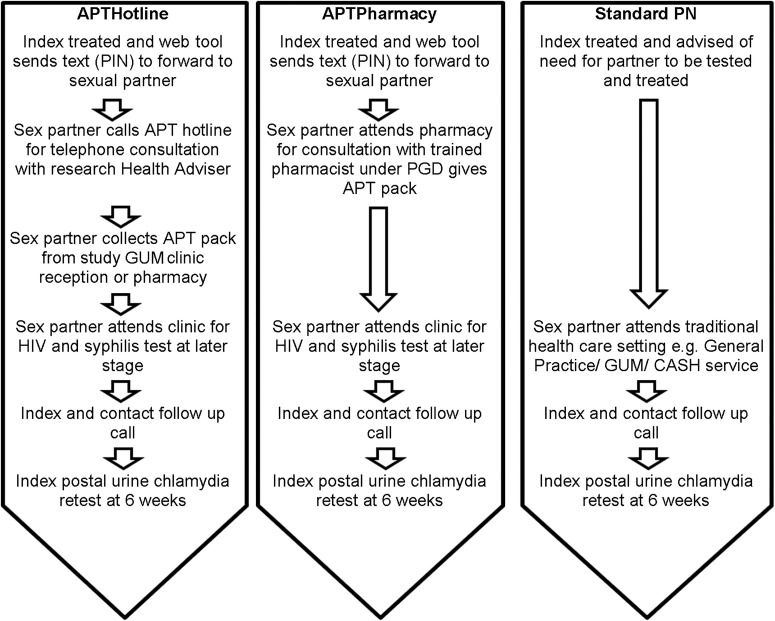
Interventions and clinical management pathways. *Accelerated partner therapy (APT) pack*—contained prepackaged azithromycin 1 g, condoms, chlamydia information leaflet, a urine sample collection kit for *Chlamydia trachomatis* nucleic acid amplification test (NAAT) with instructions to provide the sample before taking the antibiotics, prepaid postal envelope and packaging for returning the sample to the study clinic, and a patient information leaflet about the study. *APTHotline*—sex partner undergoes telephone consultation and invitation for future clinic based HIV and syphilis screening with research health adviser. Sex partner or his representative collects APT Pack from clinic reception. Sex partner posts back his completed *C. trachomatis* NAAT urine sample kit (contained in the APT pack). Results and future clinical care are managed by specialist clinic. *APTPharmacy*—sex partner undergoes consultation and invitation for future clinic-based HIV and syphilis screening with sexual health trained community pharmacist. Pharmacist gives sex partner APT pack at the time of consultation, based on a patient group direction (PGD) which is a legal framework that allows some healthcare professionals to supply a specified medicine to a predefined group of patients without a doctor assessment. Sex partner posts back his completed *C. trachomatis* NAAT urine sample kit (contained in the APT pack). Results and future clinical care are managed by specialist clinic. *Standard Partner notification (PN) (control)*. For the purposes of this study we define standard PN as the healthcare professional advising the index patient to notify his/her sex partner of the need for treatment (simple patient referral). In the contraception and sexual health (CASH) services this was supplemented by written information about chlamydia and the provision of details of local sexual health services to the index patient to give to her sex partner (enhanced patient referral). GUM, genitourinary medicine.

All index patients were offered standard PN irrespective of the arm (figure 1). Therefore, index patients randomised to APT options were offered APT *in addition* to standard PN for all of their partners. During the PN discussion with the index patient, the health professional entered data directly onto the web tool and the web tool randomised the index patient's sexual partners to one of the three arms. If an APT arm was allocated, the web tool simultaneously sent an SMS (text message) to the index patient with a unique personal identification number (PIN). The healthcare professional asked the index patient to talk to her partner(s) about their need for testing and treatment and to forward them the PIN as this was required to access APTHotline or APTPharmacy treatment. If the index patient was allocated to standard care she did not receive an SMS message.

We refined the web tool used in a previous study^9^ to support the APT patient pathway in primary care, described in detail elsewhere.^19^

### Outcome measures and ascertainment of outcomes

The primary outcome was whether each contactable partner (reported as contactable by the index at initial consultation) was treated within 6 weeks of index diagnosis. This was either partner verified by the partner calling the APTHotline or attending the APTPharmacy, or reported by the index at a follow-up telephone assessment conducted by health advisers 4–6 weeks after index treatment. Partners for whom treatment information was unavailable were considered untreated. Secondary outcome measures were determined either for the index or for each partner and included whether the partner was notified, partner uptake and acceptability of PN modalities, number of partners treated per index patient, number of partners notified per index patient, time to partner treatment and *C. trachomatis* reinfection/persistence rates in index patients. For treated partners we also report the PN method leading to treatment (standard, APTHotline, APTPharmacy, other). We also collected data on resource use to inform our health economics evaluation, reported elsewhere.^19^

To determine reinfection/persistence, 4–6 weeks after treatment we sent index patients who could be reached for telephone follow-up and who agreed a urine collection kit for *C. trachomatis* nucleic acid amplification test (NAAT) testing, for posting to the study laboratory.

### Process evaluation

We monitored recruitment processes by observing clinical staff in study sites and conducted informal interviews with a range of clinicians involved with the study. We explored issues of acceptability and preference by undertaking qualitative telephone interviews with a sample of index patients and their sex partners, on which we conducted a preliminary thematic analysis, reported elsewhere.^19^

### Sample size

The study was designed as a pilot trial to provide estimates for a definitive trial, in line with the Medical Research Council framework for development of complex interventions^20^ with randomisation of index patients on an individual basis, recognising that there would be clustering of partners by index patient. The unit of analysis was the individual male partner. We assumed that 25% of male partners would be reported (by index or healthcare service) as treated via routine PN in primary care in comparison with 40% of male partners who would be reported as treated via either of the APT options, and sought 80% power. A 2% significance level was chosen (to take account of the need to make comparisons across three arms (routine, APTHotline, APTPharmacy)), and a 10% design effect (to take account of clustering of an average of 1.5 male partners per female index patient).

We therefore aimed at recruiting 400 index patients across the two geographical areas over 24 months. However, as recruitment rates were lower than anticipated, a pragmatic decision was made during the trial to seek full outcome data (including secondary outcomes from index follow-up interviews) for at least 200 partners in total across study arms.

### Randomisation

Index patients were randomised in a 1:1:1 ratio to one of the three study arms, by simple computer-generated unrestricted randomisation within the web tool. The randomisation applied to all contactable partners identified by the index. It was not feasible for participants or researchers to be blind to the intervention type during implementation or evaluation.

### Statistical analyses

Analysis was by intention to treat and blinded. For the primary outcome, all partners not reported as treated were analysed as if untreated. Proportions of partners considered treated, considered untreated and treatment status unknown (by whether index was followed-up or not) are also reported.

We used logistic regression to calculate adjusted ORs comparing both APTHotline and APTPharmacy with routine PN. ORs were calculated adjusting for the age and ethnic group of the index patient. The analysis of outcomes determined for each partner, such as the primary outcome, was based on robust SEs to acknowledge the clustering of partners by index. All analysis was conducted in Stata V.13.

## Results

**Figure 2:**
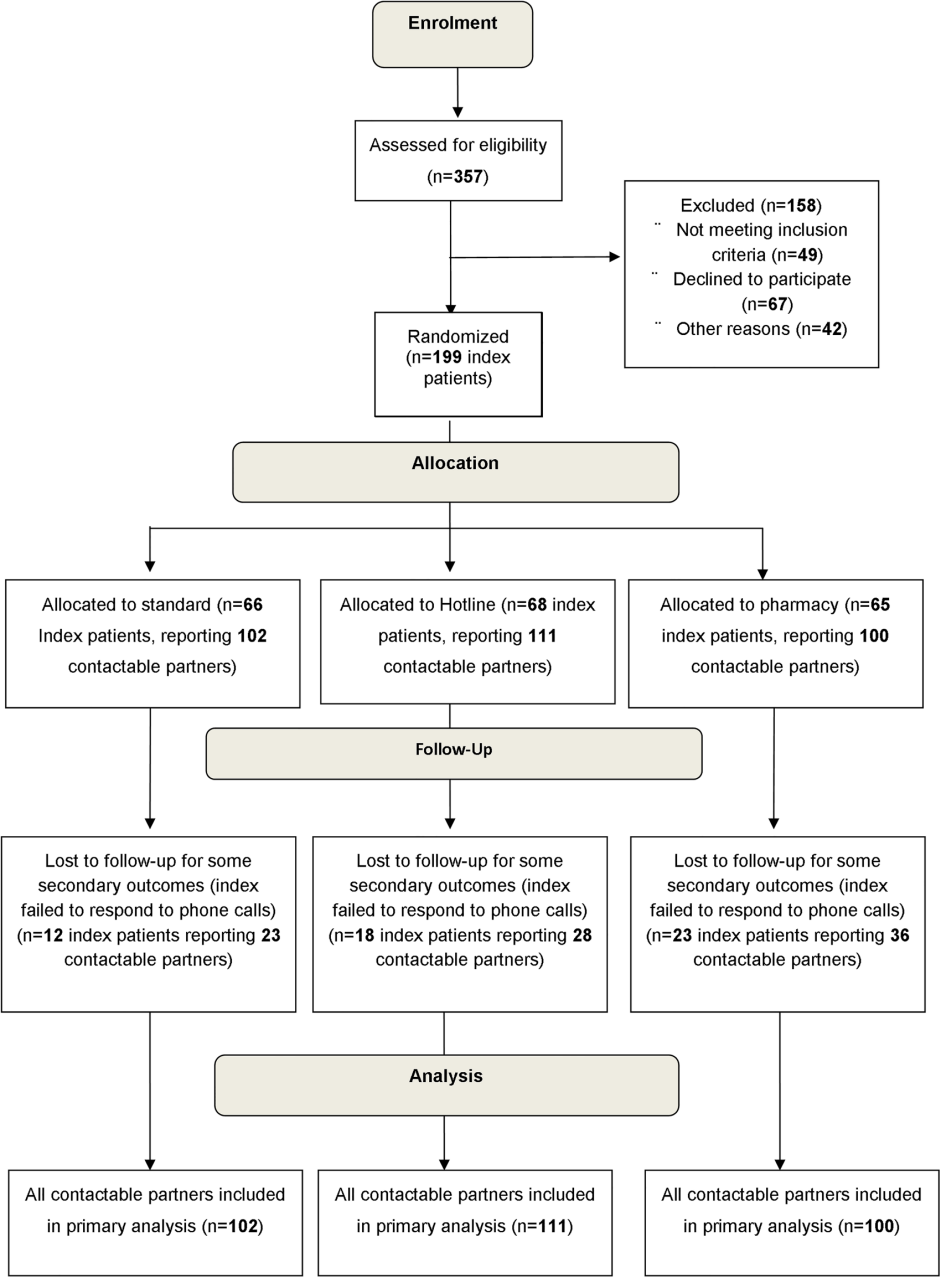
Consort diagram of participants through the trial.

Of 357 chlamydia-positive women screened for eligibility, 49 were found not to be eligible, 67 declined to participate and 42 were not included for other reasons (figure 2). A total of 199 index patients were randomised, who in total identified 313 contactable partners.

As all partners not known to be treated were considered to be untreated there was no loss to follow-up for the primary outcome. However, some secondary outcomes were obtained from the index telephone follow-up which failed to occur (index designated unreachable after three attempts) for roughly a quarter of index patients (53 of 199) and affecting 87 partners (28% of 313).

Baseline characteristics were similar across arms. The median age of index patients was 21 years, with a mean of 1.7 sexual partners over the last 6 months, of whom a mean of 1.6 were stated to be contactable (table 1).

**Table 1:** Characteristics of index patients and sexual partners by randomisation arm

Index patients				
Arm: characteristic	Standard N=66	APTHotline N=68	APTPharmacy N=65	Total N=199
Age in years, median (IQR)	20 (19–22)	21 (18–23)	21 (19–24)	21 (19–23)
Ethnicity, % (n)				
White British	59 (39)	53 (36)	57 (37)	56 (112)
White other	8 (5)	15 (10)	18 (12)	14 (27)
Mixed	2 (1)	6 (4)	8 (5)	5 (10)
Black/Black British	3 (2)	3 (2)	2 (1)	3 (5)
Asian/Asian British	11 (7)	10 (7)	6 (4)	9 (18)
Other	8 (5)	3 (2)	3 (2)	5 (9)
Number of sexual partners, % (n)				
1	64 (42)	56 (38)	60 (39)	60 (119)
2	23 (15)	22 (15)	23 (15)	23 (45)
3	8 (5)	15 (10)	9 (6)	11 (21)
4+	6 (4)	7 (5)	8 (5)	7 (14)
Mean (SD)	1.6 (1.18)	1.8 (1.10)	1.7 (1.12)	1.7 (1.13)
Number of contactable sexual partners, % (n)				
1	71 (47)	62 (42)	68 (44)	67 (133)
2	17 (11)	19 (13)	15 (10)	17 (34)
3	6 (4)	15 (10)	12 (8)	11 (22)
4+	6 (4)	4 (3)	5 (3)	5 (10)
Mean (SD)	1.5 (1.18)	1.6 (0.94)	1.5 (0.89)	1.6 (1.01)
Contactable partners				
Arm	Standard N=102	APTHotline N=111	APTPharmacy N=100	Total N=313
Relationship to the index, %(n)				
Cohabiting/married/civil partnership	3 (3)	14 (15)	7 (7)	8 (25)
Steady but not cohabiting	48 (49)	38 (42)	44 (44)	43 (135)
Sex once	30 (31)	23 (26)	27 (27)	27 (84)
Have sex from time to time	3 (3)	12 (13)	12 (12)	9 (28)
Ex-partner	12 (12)	8 (9)	6 (6)	9 (27)
Just met for the first time	4 (4)	5 (6)	4 (4)	4 (14)

APT, accelerated partner therapy.

The proportion of partners known to have been treated varied little between arms (39% APTHotline and 46% in each other arm) (table 2) and there was little evidence of difference between APT intervention and standard patient referral. The slightly lower proportion known treated in the APTHotline arm may reflect a higher proportion for whom treatment status was unknown (60% vs 44% Standard and 47% APTPharmacy). The mean number (SD) of sexual partners known treated per index by randomisation arm was Standard 0.7 (0.58), APTHotline 0.6 (0.74), APTPharmacy 0.7 (0.70). Among partners randomised to APTHotline, only 21% were treated via the hotline, and of those randomised to APTPharmacy, 30% were treated via a pharmacy (table 2).

**Table 2:** Treatment and notification of contactable partners by randomisation arm

	Standard N=102 % (n)	APTHotline N=111 % (n)	AOR* (95% CI)	APTPharmacy N=100 % (n)	AOR* (95% CI)	Total N=313 % (n)
Notification						
Notified	74 (75)	68 (75)	0.91 (0.48–1.73)	66 (66)	0.90 (0.65–1.27)	69 (216)
Not notified	4 (4)	11 (12)		3 (3)		6 (19)
Unknown (index followed-up)	0 (0)	0 (0)		0 (0)		0 (0)
Unknown (index not followed-up)	23 (23)	22 (24)		33 (33)		25 (78)
Treatment						
Treated	45 (46)	35 (39)	0.64 (0.35–1.18)	46 (46)	1.06 (0.78–1.45)	42 (131)
Not treated	11 (11)	5 (5)		7 (7)		7 (23)
Unknown (index followed-up)	22 (22)	37 (41)		15 (15)		25 (78)
Unknown (index not followed-up)	23 (23)	23 (26)		32 (32)		26 (81)
Treated partners only, %(n)	Standard, N=46	Hotline, N=39		Pharmacy, N=46		Total, N=131
Location of treatment						
APTHotline	N/A	21 (8)		N/A		6 (8)
APTPharmacy	N/A	N/A		30 (14)		11 (14)
GP	28 (13)	21 (8)		7 (3)		18 (24)
GUM clinic	46 (21)	26 (10)		35 (16)		36 (47)
Other	11 (5)	8 (3)		9 (4)		9 (12)
Unknown (Index followed-up)	15 (7)	26 (10)		20 (9)		20 (26)

*OR for partner notified relative to any other outcome**,** adjusted for index age and ethnicity, relative to standard arm.

APT, accelerated partner therapy; GP, general practitioner; GUM, genitourinary medicine.

### Secondary outcomes

#### Time to partner treatment

The median time (IQR) from index diagnosis to partner treatment was 0 (0–0 days) for routine PN, 0 (0–0 days) for APTHotline (p=0.78) compared with routine and 0 (0–4 days) for APTPharmacy (p=0.02) compared with routine.

#### Index patient reinfection/persistence

Only 38/199 index patients (19% of total) returned a postal urine sample for reinfection/persistence and chlamydia positivity was 15% (2/13) in the standard arm, 0% (0/15) in the hotline arm and 10% (1/10) in the pharmacy arm.

#### Sex partner testing

In the standard arm no partners were reported to have had a chlamydia or gonorrhoea test by the index patients, compared with 4% (4/111) in the APTHotline arm (one of whom had a positive chlamydia test) and 6% (6/100) in the APTPharmacy arm. No partners were known to have attended a clinic for an HIV or syphilis test.

### Process evaluation

Community healthcare professionals found the web tool easy to use as it provided a user-friendly interface on which to document the PN discussion. However, seeking informed consent at an emotionally difficult time for the index patient was perceived to be difficult and in some cases led to healthcare professionals failing to offer the study.

Although all services were highly supportive of the research, they experienced difficulty implementing the study and recruitment was suboptimal. Both CASH services underwent major organisational changes and all the general practices were affected by wide-ranging national changes in service commissioning during the recruitment period and research was not prioritised. We centralised recruitment by enabling the research health adviser to offer the study to eligible patients by telephone. This resulted in a rapid increase in recruitment rates.

As regards acceptability to participants, follow-up interviews with index patients and sex partners showed high levels of acceptability of the APT interventions.^19^ No harmful clinical incidents were reported.

## Conclusions

We developed and tested two novel replicable, feasible, acceptable and safe APT models for use in primary care settings, which met General Medical Council guidance^8^ on remote prescribing. However, individual recruitment to the trial was difficult. Overall, a similar proportion of partners were reported as treated in all three arms of the trial, in each case fewer than half, and the addition of APT interventions did not appear to improve outcomes. Uptake of the adjunct interventions was poor and most PN was achieved via standard methods, rather than via APT. The online patient and data management tool was acceptable to clinical staff and feasible for the referral of patients for PN support and outcome measurement.

Women reported numbers of sex partners comparable to women attending specialist sexual health services^9^ and the majority of sex partners were contactable. No sex partners reported receiving HIV and syphilis testing, though a small number of sex partners in APT arms returned a postal test kit for chlamydia and gonorrhoea. Despite good levels of index patient follow-up, return of postal test kits for reinfection testing 6 weeks post treatment was low.

Achieving effective PN in primary care is problematic^11^ ^13^ ^14^ ^21^ but the importance of improving the effectiveness of PN as a strategy for improving population control of *C. trachomatis* is well recognised.^7^ There are challenges to the comparison of our data with other studies. We report PN outcomes primarily per partner (although we include outcomes per index patient), as we feel that this is a helpful measure of the potential for prevention of further transmission.^22^ However, current guidelines for PN, and newly available surveillance reports in England, continue to focus on outcomes per index patient.^23^ Nevertheless, the suboptimal rates of PN described here are superior to those reported in similar settings.^11^ ^13^ ^23^

Historically, non-specialist settings have not had a routinely available PN infrastructure and PN outcomes have rarely been measured or recorded. The provision of a formal PN service and the presence of researchers are both likely to produce a substantial ‘Hawthorne effect’^24^ by normalising the discussion of PN. This may alone empower an index patient to achieve patient referral.

Our previous study of APT in specialist sexual health settings used partner focused outcome measures and suggested that APT could improve PN outcomes as compared with standard patient referral.^9^ ^10^ However, there were important differences in the design, inclusion criteria and exact nature of the interventions which make direct comparisons difficult. Our earlier study allowed index patients to select the method of PN they thought would best suit each partner whereas this study randomised patients. In routine clinical practice, index patients choose from a range of PN options and we believe that randomising patients to a single intervention may not be the optimal design for sensitive interventions such as PN.^25^

Here, time to sex partner treatment was similar across all arms, whereas in our previous APT study, sex partners in APT arms achieved treatment faster than those receiving standard PN.^9^ This difference is likely to be explained by poor uptake of APT interventions within the intervention arms.

Implementation of a pilot trial of this size, with multiple recruitment sites, at a time of wide ranging health service upheaval^26^ was extremely difficult. We had to reduce our original sample size to provide more realistic recruitment requirements for each service and support recruitment centrally. Our primary outcome was largely index reported rather than sex partner verified, which introduces uncertainty around the robustness of outcome ascertainment. Indexes may not have known whether their sex partners received STI testing as well as treatment. ‘Standard PN’ could be enhanced or simple patient referral depending on the service and or the healthcare professional involved. This may have made it hard to demonstrate an added benefit of the APT interventions.

Although we included a biological outcome, in practice, it did not prove useful as index postal retesting had low uptake despite telephone reminders. We do not know whether the partners of women who declined to take part were more or less likely to be treated. Our sample could therefore be biased in either direction with respect to the target population of index patients and partners.

The care pathways we developed, using a novel online patient referral and data management tool, provide a feasible and acceptable infrastructure for onward referral of patients diagnosed with STIs in primary care, to receive PN support. These could usefully form part of a portfolio of PN modalities which collectively improve overall PN outcomes. The low uptake of STI testing and HIV testing is notable, and suggests that modes of PN which do not require direct engagement with a clinical service offering comprehensive testing may be unsuitable for higher risk populations. While in the future this could be addressed through postal self-sampling for a wider range of infections, they are not suitable either for individuals or for populations who have been identified as higher risk, such as men who have sex with men (MSM) and MSM partners of heterosexual women.^27^

Overall, PN outcomes were superior to previously reported PN measures in similar settings but an in-depth understanding of reasons for poor uptake of the interventions in this study will be important for future PN research irrespective of the setting. A cluster, non-consented (Zelen) design in which whole services are randomised to the offer of an APT intervention is likely to circumvent many of the difficulties faced by this trial. Although controversial, this approach has been undertaken in the field of chlamydia screening.^28^ ^29^ Research should also be undertaken to explore how to identify and reach higher risk partners in primary care settings who may benefit from a more comprehensive range of sexual health services.

**Key messages**

- We developed two novel primary care accelerated partner therapy (APT) models, showing them to be acceptable, feasible, regulation compliant and capable of achieving acceptable outcomes within a pilot randomised controlled trial (RCT).
- Addition of these interventions to standard partner notification (PN) did not result in a difference between arms, but overall PN uptake was higher than previously reported in similar settings.
- Recruitment to an individually randomised trial proved challenging and full evaluation is likely to require service-level randomisation.
